# P-2186. Effects of Knockdown of DDX58 Gene on EV-A71 Replication and Regulation of the RIG-I Receptor Pathway

**DOI:** 10.1093/ofid/ofaf695.2349

**Published:** 2026-01-11

**Authors:** Guoe Gou, Meng Zhang, Mei Li, Muqi Wang, Wen Zhang, Song Zhai, Chenrui Liu, Ting Li, Yaping Li

**Affiliations:** The Second Affiliated Hospital of Xi'an Jiaotong University, Xi'an, Shaanxi, China; The Second Affiliated Hospital of Xi'an Jiaotong University, Xi'an, Shaanxi, China; The Second Affiliated Hospital of Xi'an Jiaotong University, Xi'an, Shaanxi, China; The Second Affiliated Hospital of Xi'an Jiaotong University, Xi'an, Shaanxi, China; Xi'an Jiaotong University Second Affiliated Hospital, Xi'an, Shaanxi, China; Second Hospital of Xi'an Jiaotong University, Xi'an, Shaanxi, China; Xi'an Jiaotong University Second Affiliated Hospital, Xi'an, Shaanxi, China; The Second Affiliated Hospital of Xi'an Jiaotong University, Xi'an, Shaanxi, China; Xi'an Jiaotong University Second Affiliated Hospital, Xi'an, Shaanxi, China

## Abstract

**Background:**

This study aimed to investigate the impact of DDX58 knockdown on EV-A71 replication and the activation of the RIG-I-like signaling pathway, and to analyze its role in antiviral immune responses.

**Methods:**

DDX58 knockdown was achieved using siRNA transfection in RD cells, followed by infection with EV-A71. mRNA and protein levels of EV-A71, DDX58, and key downstream molecules in the RIG-I pathway were analyzed by qRT-PCR and Western blotting. The concentrations of interferons (IFNα, IFNβ) and CXCL-10 were determined by ELISA.

**Results:**

Knockdown of DDX58 significantly inhibited EV-A71 replication, as evidenced by a reduction in both EV-A71 mRNA and VP1 protein levels. Additionally, the expression of key antiviral signaling molecules, including IRF3, IRF7, and CXCL-10, was upregulated at 24 hours post-infection, but decreased at 36 hours. Notably, the concentrations of IFNα and IFNβ were elevated at 24 hours but showed a decline at later time points.

**Conclusion:**

DDX58 plays a critical role in modulating EV-A71 replication and immune responses, with a complex bidirectional regulatory effect on the RIG-I pathway. These findings suggest that DDX58 could be a potential therapeutic target for regulating antiviral immune responses in EV-A71 infection.

**Disclosures:**

All Authors: No reported disclosures

